# Metabolic modeling of host-microbe interactions

**DOI:** 10.1016/j.csbj.2025.10.006

**Published:** 2025-10-03

**Authors:** Natchapon Srinak, Florian Krüger, Christoph Kaleta, Jan Taubenheim

**Affiliations:** Research Group Medical Systems Biology, Institute of Experimental Medicine, Kiel University and University Hospital Schleswig-Holstein, Kiel, Germany

**Keywords:** Multi-species modeling, Host-microbe interactions, Community modeling, Genome scale metabolic modeling, Constrained-based reconstruction and analysis (COBRA)

## Abstract

Host-microbe interactions play an integral role in the function and survival of eukaryotes, influencing various processes ranging from metabolism to immune regulation. As our understanding of these interactions deepens, there is a growing shift toward integrative approaches that consider both host and microbial genotypic potential. However, capturing the complexity and dynamic nature of these relationships remains a significant challenge. Genome-scale metabolic models (GEMs) offer a powerful framework to investigate host-microbe interactions at a systems level. By simulating metabolic fluxes and cross-feeding relationships, GEMs enable the exploration of metabolic interdependencies and emergent community functions. These models can be applied independently or in conjunction with experimental data, supporting hypothesis generation and systems-level insights into host-microbe dynamics. In this review, we examine recent applications of GEMs to host-microbe studies, with a focus on how they reveal reciprocal metabolic influences. We also discuss the current technical challenges, highlight available tools and methodological strategies, such as model reconstruction, data integration, and simulation and analysis steps, and outline future directions for advancing host-microbe interaction study using GEMs.

## Graphical Abstract

**Figure:**
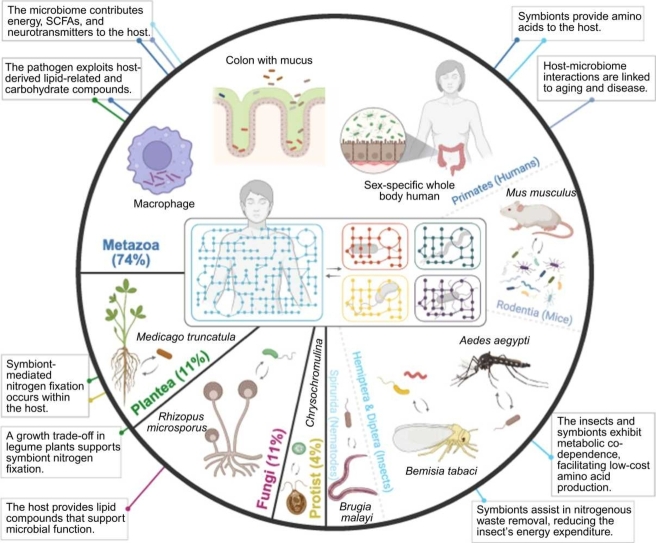

## 1 Introduction

All eukaryotic host organisms are colonized by microbial communities, numerous species of microorganisms including bacteria, archaea, fungi, and viruses coexisting within a specific environment [1], [2]. Their coexistence results in reciprocal adaptations or co-evolution leading to intricate interactions between the host genome and the microbiome, denoting all microbial genomes in the microbial community [3], [4]. Many studies have highlighted that microbial communities can beneficially influence host metabolism, development, immunity, and even behavior [5], [6], [7], [8]. Their collective function emerges from complex interactions among the microbes themselves and surrounding environments. In turn, the host exerts mechanistic control over the microbial community, actively maintaining communication with microbes by shaping environmental niches. For example, the host regulates nutrient availability, immune responses, and habitat conditions, enabling a functional microbial ecosystem [8], [9], [10], [11]. These mechanisms help stabilize microbial composition; however, when host control is disrupted - whether due to genetics or regulatory deficiency of the host itself or environmental stressors - the microbial community's structure and function may be disturbed, a state known as dysbiosis [12]. Such microbial dysbiosis can negatively impact host fitness and therefore the host and its associated microbes are thought to form a unit of selection during evolution, a concept known as holobiont or metaorganism [13], [14], [15], [16], [17], [18]. The system is highly complex and often dynamic, making it challenging to accurately represent its ecosystem holistically and mechanistically in response to environmental perturbations.

While reductionistic approaches provide valuable insights into host-microbe interactions, they are inherently limited in capturing the complexity of natural ecosystems and are often restricted in their scalability [19], [20]. Empirical experiments using host cells, organoids, or organ-on-a-chip models allow for the testing of the effect of specific microbial products on host cells; however, they are only able to capture isolated effects rather than the full complexity of host-microbe interactions. In contrast, in vivo models - for instance gnotobiotic vs. wild-type or humanized models (using fecal microbiota transplantation (FMT)), offer a more comprehensive understanding of host-microbe interactions. Yet, conducting functional tests and pinpointing individual factors which contribute to the observed effects can be difficult. The generation of multi-omic data, such as metabolomics, proteomics, metagenomics, and metatranscriptomics from clinical samples and animal models, has further enhanced our understanding of host-mediated microbial regulation and microbiota effects on host fitness [9], [21]. Although these methods provide broad insights into host-microbe interactions, they often lack the spatial resolution (e.g., directionality of cell-to-cell interactions) and temporal dynamics (e.g., time-dependent effects and intensity level of environmental influences on cellular behavior) necessary for unraveling the intricate interactions like metabolic cross-feeding that are critical to host-microbe ecology. Labeling experiments, such as ¹ ³C and ¹ ³N metabolic flux analysis, can capture detailed interactions between hosts and microbes, as well as microbe-microbe interactions [22], [23], [24], however, these techniques are typically performed in controlled synthetic environments, limiting their ability to replicate the complex interactions observed in natural or multi-species communities. The inherent complexity of such systems, combined with experimental constraints, hampers our ability to fully characterize host-microbe interactions, restricting the development of more precise applications in fields such as medicine, agriculture, husbandry, and ecology.

Advances in next generation sequencing technologies and systems biology have facilitated a shift from single-species studies to more comprehensive, interkingdom analyses. One approach supporting the study of interkingdom interactions is genome-scale metabolic modeling (GEM), which enables the integration of multi-species microbial data with host genomic information, paving the way for a deeper understanding of these complex systems on a metabolic level [25]. A GEM is a mathematical representation of the metabolic network of an organism, based on its genome annotation. It includes a comprehensive set of biochemical reactions, metabolites, and enzymes that describe the organism's metabolic capabilities [25]. Using computational tools, GEMs of different species can be combined to an integrated model and used to depict metabolite flow between hosts and microbes, providing valuable insights into their interactions [26], [27]. Here, we review the applications of GEMs in the context of host-microbe interactions, with a particular emphasis on the integration of host and microbe models within diverse host systems. We emphasize the ability of host-microbe GEMs to uncover the intricate reciprocal influences between hosts and microbes, while also discussing the technical implementations, key challenges, and future directions of this approach.

## 2 Technical and computational considerations in host-microbe modeling

Metabolic modeling is predominantly performed within the framework of constrained-based reconstruction and analysis (COBRA) [28]. Here a model is represented as a stoichiometric matrix, which depicts the stoichiometric relationship, e.g. the production and consumption (values) of metabolites (rows) and reactions (columns) [29]. A key analysis tool in COBRA is flux balance analysis (FBA) to estimate flux through reactions in the metabolic network [30]. Assuming that most metabolism happens in steady states, FBA ensures that the total flux of metabolites into an internal reaction equals outflux (mathematically S*v=0, with S being the stoichiometric matrix and v the flux vector). Afterwards, FBA tries to optimize the flux vector through the GEM to fulfil a defined aim, the objective function (usually maximum biomass production). The assumption of steady state transforms the enzyme kinetics into linear problems where output only depends on input, hence the problem can be solved using linear programming solvers like GLPK, Gurobi or CPLEX. However, the resulting solution is usually just one of many possible flux distributions through the GEM which could lead to the same optimal value in the objective function. Hence, it is important to constrain the model as much as possible, by including additional information. This usually includes (in-) active reactions, expected flux ranges of reactions and the expected nutritional environment (the medium or diet). Furthermore, it is common to minimize the total flux through the model, to ensure the most efficient flux distribution to achieve maximum growth [31]. These measures ensure that flux distributions are realistic and less variable in different modeling scenarios. Current trends tend to add additional information, like reaction rates and protein abundance to constrain metabolic models and have been extensively reviewed elsewhere [32].

### 2.1 Model reconstruction and constraints

**Fig. 1:**
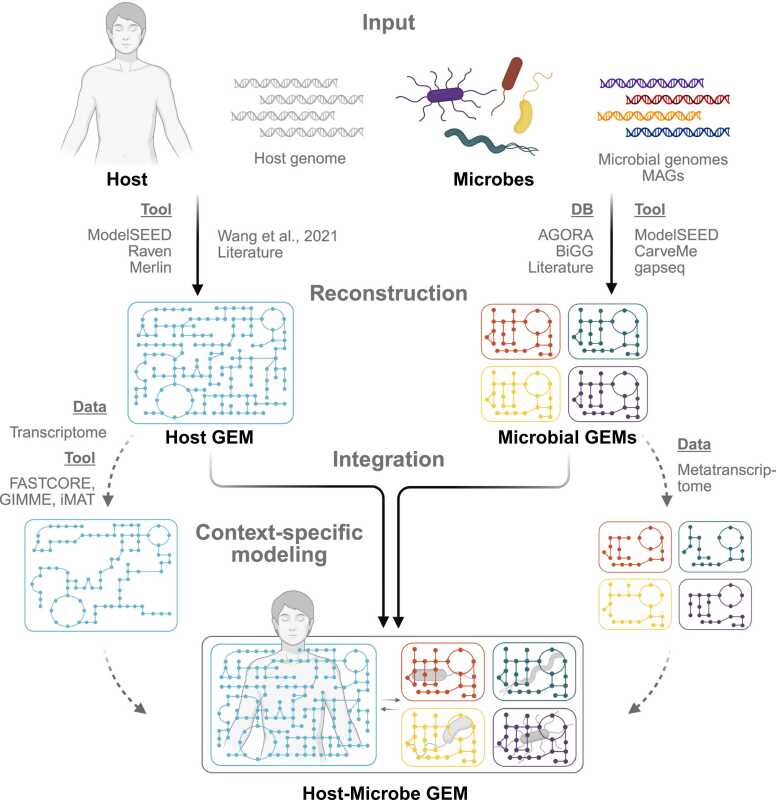
Development of host-microbe GEMs. Genomes of the host and microbes, including metagenome-assembled genomes (MAGs) for microbes, serve as input for genome-scale metabolic model (GEM) reconstruction. Automated reconstruction tools, databases, published high-quality GEMs, and standardized guidelines facilitate either de novo reconstruction from genomes or direct retrieval of existing GEMs. These models can then undergo context-specific refinement using condition-dependent data (e.g., transcriptome or metatranscriptome) or be directly integrated into the host-microbe GEM. DB - database.

The development of host-microbe GEMs typically involves three main steps: (i) collection or generation of input data for both host and microbial species, e.g., genome sequences, metagenome-assembled genomes, and physiological data; (ii) reconstruction or retrieval of individual metabolic models using curated databases, literatures, or automated pipelines; and (iii) integration of these models into a unified computational framework (Fig. 1). Developing a host-microbe metabolic model presents several challenges, particularly in the reconstruction and integration of host and microbial models, for which previous studies have employed different strategies (Table S1). Microbial metabolic models are relatively easier to derive due to the availability of high-quality GEMs repositories such as AGORA [33], [34], BiGG [35], and APOLLO [36], which provide well-curated models for various microbial species. Additionally, automated metabolic model reconstruction tools like ModelSEED [37], [38], CarveMe [39], gapseq [40], and RAVEN [41] facilitate the rapid generation of microbial models directly from genomic data. In contrast, reconstructing a host metabolic model, particularly for eukaryotic cells, is more complex. Several factors contribute to this complexity, including incomplete genome annotations, the precise definition of biomass composition, and the compartmentalization of metabolic processes, for example, in mitochondria, peroxisomes, and endoplasmic reticulum [42], [43]. Multicellular eukaryotes show further compartmentalization on the cell level, where specific metabolic tasks are performed by specialized cells, further complicating the metabolic network reconstruction. For eukaryotes, tools like ModelSEED (PlantSEED) [37], [38], [44], RAVEN [41], [45], merlin [46], [47], AuReMe [48], and AlphaGEM [49] have been used to reconstruct draft models from genome sequences, including models for plants, algae, fungi, and parasites. Unfortunately, these models require extensive refinement and manual curation to ensure biological accuracy and remove thermodynamic infeasible reactions. High-quality host models are typically developed through a semi-manual or manual approach [50], [51], where reactions and pathways are systematically curated based on existing knowledge e.g., the high-quality human metabolic model, Recon3D [52]. Additionally, published GEMs for various eukaryotic hosts, such as *Saccharomyces cerevisiae* (yeast) [53], *Arabidopsis thaliana* (Arabidopsis) [54], and *Mus musculus* (mouse) [55], could serve as valuable resources for model refinement and development. Once both host and microbial models are reconstructed, integrating them into a unified framework presents another challenge. These models are often derived from different sources, using distinct nomenclatures for metabolites, reactions, and genes. This makes the integration between models difficult. Standardization efforts such as MetaNetX provide a unified namespace for metabolic model components, helping to bridge discrepancies between different sources [56]. Despite this resource, the lack of standardized formats and model integration pipelines remains a critical bottleneck in host-microbe modeling. Thus, automated approaches for harmonizing, and merging models from diverse sources are needed to support the development of integrated models of host and microbiota. This includes the detection and removal of thermodynamically infeasible reactions which create free energy metabolites or resources in the integrated model. These are often introduced by merging models of different origin for example due to inconsistencies of protonation states or different number of units in polymeric compounds. To avoid unrealistic model results, these reaction cycles have to be detected and corrected during the merging process.

Apart from the reconstruction of genome-scale models, context-specific metabolic modeling offers advantages in studying condition-dependent metabolic fluxes [57]. These models integrate omics data as an additional layer of metabolic regulation by constraining the models further to act within the borders of “experimental context” and enable a precise understanding of metabolic activity under specific physiological states or environmental conditions. Several tools, including INIT [58], iMAT [59], mCADRE [60], FASTCORE [61], GIMME [62], and GIM³E [63] have been developed to incorporate multi-omics data, such as (meta-) transcriptomics, proteomics, and metabolomics for refining metabolic models based on experimental conditions. Moreover, first attempts in single cell metabolic model reconstruction have been made by using single cell transcriptomics for contextualization [64], [65]. These data integration efforts have great value in understanding metabolic regulation during host-microbe interactions. For example, recent work integrating transcriptomic data into a single species metabolic model of *Caenorhabditis elegans*, revealed that host sphingolipid and fatty acid metabolism shifts depending on the interacting microbial strains [66]. Metatranscriptomic data integration for microbial community metabolic models, revealed a link between reduced short chain fatty acid (SCFA) exchange and Crohn’s disease [67]. However, applying these approaches to host-microbe metabolic models requires experimentally and analytically challenging procedures. For instance, co-isolating microbial and host mRNA leads to several challenges such as imbalance of host-microbe sequencing depth that usually causes underrepresentation of microbial RNA. Further, the sheer amount of data demands higher computing facilities which can become a bottleneck in analyzing large amounts of samples [68]. Although these advanced experimental techniques and bioinformatics methods have been developed to retrieve and analyze these data simultaneously [69], [70], [71], extracting context-specific metabolic models from these host-microbiome-omics data has been so far reported in only one study [72] and remains subject of ongoing research.

### 2.2 Model simulation and analysis

In GEM simulations, FBA is a widely used computational approach that employs linear programming to optimize metabolic fluxes under the steady-state assumption [25], [30]. Once simulated, the GEM provides readily interpretable results in the form of flux distribution. The flux values (typically expressed in ${\mathrm{mmol}\cdot h}^{-1}\cdot g_{\mathrm{dry weight}}^{-1}$) are directional by definition. This allows exchange metabolites to be directly attributed to their compartment of origin. Typically, FBA maximizes or minimizes an objective function to predict cellular behavior within a set of biological constraints. In single-species models, this objective function often corresponds to the maximization of biomass or ATP production, based on the assumption that microorganisms prioritize growth and reproduction [73]. In multi-species microbial models, relative abundance data from 16S rRNA or shotgun metagenome sequencing are frequently used as proxies for relative biomass biosynthesis rates. These values are incorporated into the community biomass equation as stoichiometric coefficients, attempting to model species interactions while optimizing community growth [74]. However, optimizing solely for community biomass implicitly assumes perfect cooperation among individual species, which may not reflect biological reality. To address potentially competing goals of individual species and the whole bacterial community, bi-level optimizations can be used where the growth of individual species is balanced with a separate optimization for the community. This optimization can still be community growth, as implemented in OptCom [75] or CASINO [76], but the concurrent growth optimization for individual species prevents unrealistic altruistic behavior. Alternatively, approaches like NECom [77] forgo optimization of direct objective functions for the community. Instead, they derive steady-state community solutions based on pure-strategy Nash equilibria that reflect stable, competing metabolic strategies among species. In this context, a Nash equilibrium describes a stable state in a microbial community where each species has chosen its metabolic strategy, involving metabolite exchanges and utilization. At equilibrium, no species can change its own metabolic fluxes to grow faster without affecting or being affected by the others. Another modeling framework implements tradeoff optimizations in the tool MICOM [78]. In a two step optimization MICOM optimizes first overall community growth, afterwards individual growth and an optimal tradeoff between both is probed. Finally, agent based metabolic frameworks exist, where individual bacterial growth is optimized and FBA is applied in distinct time steps, simulating microbial interaction dynamics across time and space [79], [80]. All these frameworks vary in their initial assumptions, complexity to set them up, and computational demands, requiring in-depth considerations when choosing a tool for microbial community modeling. Table S2 summarizes the key concepts of microbial community simulation frameworks, and a more comprehensive discussion can be found in previous reviews [81], [82].

**Fig. 2:**
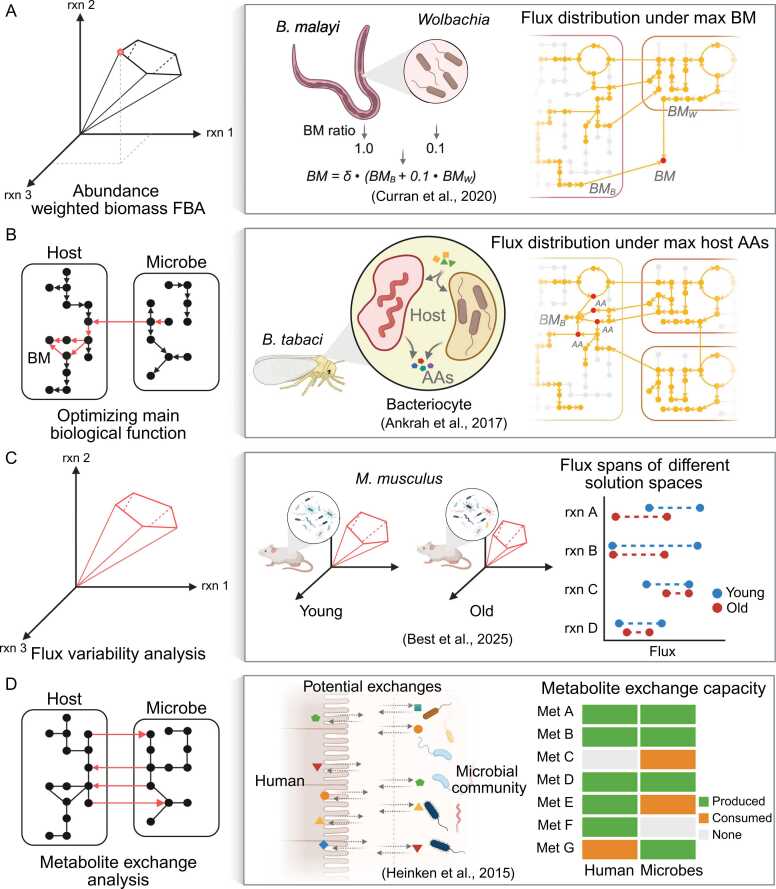
Core simulation and analysis approaches for host-microbe GEMs. (A) In well-studied systems where both host and microbial biomass compositions are known, flux balance analysis (FBA) can be used to optimize an abundance weighted biomass objective function. (B) In systems with established host-microbe metabolic interactions, specific metabolic relationships can be defined as the objective function for FBA. In less characterized systems, exploratory network analyses such as flux variability analysis (FVA) (C) or metabolite exchange analysis (D) are used. rxn - reaction, BM - Biomass, AA - amino acid.

Analogously, host-microbe modeling faces challenges in defining suitable objective functions due to differing cellular priorities: microbes typically maximize growth, whereas host cells primarily maintain metabolic homeostasis rather than proliferate, further complicating integrated model optimization. However, there are alternative simulation techniques and analytical approaches that have been used to infer metabolic interactions and explore host-microbe dependencies although the cellular objectives may not fully be known (Table 1). The choice of these approaches is guided less by prediction performance, but rather by the underlying biological questions, hypotheses, or the availability of prior knowledge and data. One approach is an abundance weighted biomass equation (similar to coupling based microbial community modeling approaches) (Fig. 2A), where well-characterized systems, such as nodule bacteria in plant roots or *Brugia malayi* and *Wolbachia*, enable the formulation of biomass equations incorporating both host and microbial contributions [83], [84]. This method relies on prior experimental data to assign weights to different organismal compartments representing their relative contribution to overall biomass in the system while maximizing overall growth. In other cases, biological knowledge about the system is utilized to formulate a specific objective function like the provision of a certain metabolite to the symbiont (Fig. 2B). For example in nitrogen-fixing bacteria-microalgae symbioses and amino acid-producing symbionts in insects, the objective function was set to maximize the production of key metabolites since experiments have shown that they are crucial for host survival [85], [86]. While this approach focused on optimizing a single metabolic function, the steady-state constraints of FBA and the connected metabolic networks inherently enforce interactions of hosts and microbes. This type of objective has been termed “hypothesis-driven objective function” as it interrogates the metabolic network in the requirements to fulfill a specific (desired) task [87]. The predicted flux distributions depend heavily on this objective function and the linked assumptions, and since FBA optimization is characterized by the most extreme solution, results are often more extreme than typical physiological conditions. Beyond predefined objectives, host-microbe interactions can be derived from exploratory analyses that provide a more flexible framework for studying metabolic interactions. Flux variability analysis (FVA), for instance, examines the range of possible metabolic fluxes within a network under different conditions (Fig. 2C), making it particularly useful for comparative studies where host-microbe interactions may shift in response to environmental changes [88], [89]. Furthermore, a key advantage of the FVA approach is that it does not rely on a specific objective function, yet it can still be used to explore the network’s metabolic capabilities based on its structure. Although constraints are not strictly required for the analysis, including key constraints, such as organism growth rates, environmental conditions, and the relative abundance of microbes, is crucial to obtain biologically meaningful and reasonable results. For example, a work recently applied FVA to quantify flux spans in young and old mouse-microbe systems, which compared these flux ranges highlighted interactions associated with the studied conditions [89] (Fig. 2C). Similarly, uptake and export potential between the host and microbe can be evaluated by maximizing the utilization and secretion of each transportable metabolite [26], [90] (Fig. 2D). The approach can reveal key metabolic dependencies between organisms based on their metabolic network and surrounding environment. The metabolite exchange analysis assumes that the exchange reactions included in the joint host-microbe models are biologically meaningful. In practice, however, some exchange reactions may be gap-filled during model reconstruction without direct genetic evidence. Therefore, interpretation of the results requires careful consideration of this limitation. Previous studies have demonstrated the use of this approach by identifying potential metabolite exchanges across the human-lumen and microbe-lumen interfaces [26].

**Table 1:** Summary of simulation and analysis approaches for host-microbe metabolic modeling.

**Approaches**	**Main assumption**	**Expected output**	**Potential software**
*Abundance weighted biomass FBA:* Incorporates existing knowledge of host-microbe biomass ratios, or proceeds (with prior knowledge) under explicit assumptions regarding the biomass ratios of the organisms.	Host and microbial biomass contributions can be weighted to represent the system’s metabolism.	Flux distribution	FBA via COBRA toolbox/cobrapy
*Optimizing main biological function:* Uses prior knowledge of the system (e.g., key functions in a symbiosis) to define the objective function for simulation.	The system’s behavior can be represented by maximizing a relevant specific biological function.	Flux distribution	FBA via COBRA toolbox/cobrapy
*Flux variability analysis:* Explores the minimum and maximum possible fluxes of each reaction under the given system's metabolic network constraints and topology.	The system’s behavior can be represented by identifying ranges of reaction fluxes under network constraints and topology.	Flux spans	FVA via COBRA toolbox/ cobrapy
*Metabolite exchange analysis:* Systematically optimizes fluxes through exchange reactions at connected compartments to evaluate the potential interactions.	All biologically important exchange reactions are present and have realistic bounds (uptake/secretion limits, permeability).	Potential exchangeable metabolites	FBA via COBRA toolbox/cobrapy
*Flux sampling analysis:* Randomly explores the feasible solution space of a metabolic network, generating distributions of possible fluxes under given network constraints and topology.	Probability of feasible metabolic fluxes follows a predefined prior (e.g., uniform). Iterative sampling converges and explores the feasible solution space sufficiently to represent all biologically relevant flux states.	Flux spans (probability)	OptGPSampler via cobrapy, CHRR sampler via COBRA toolbox, ACHR sampler via COBRA toolbox/cobrapy
*Pareto front analysis:* Identifies trade-offs between two or more biological objectives (e.g., growth maintenance of host and growth rate of a microbe) by exploring optimal flux distributions across the metabolic networks.	Interactions exist directly between a certain microbe(s) and host.	Bi-objective (multi-) trade-off and associated flux distributions	mocbapy
*Agent-based modeling FBA:* Simulates individual organisms as agents that interact in 2D space and time, with each agent’s metabolism, reproduction, or death modeled using FBA in interaction with the surrounding environment.	Organism’s growth objectives are highly independent. Metabolic interactions and spatial movements can be represented by a 2D grid and defined parameters.	Dynamic spatial and temporal arrangement, individual growth and flux distribution	BacArena

**Note:** The Main assumption column lists the distinct, explicit assumptions specific to each approach. General assumptions, such as the steady-state assumption inherited from FBA, are not included in the table. The Expected output column provides the unique result obtained from each approach. For some approaches, flux distributions are also derived; however, these are omitted from the table since they are typically considered secondary results in this context. The Potential software column provides examples of tools that can be used to implement each approach, but other suitable software may also be available.

**Fig. 3:**
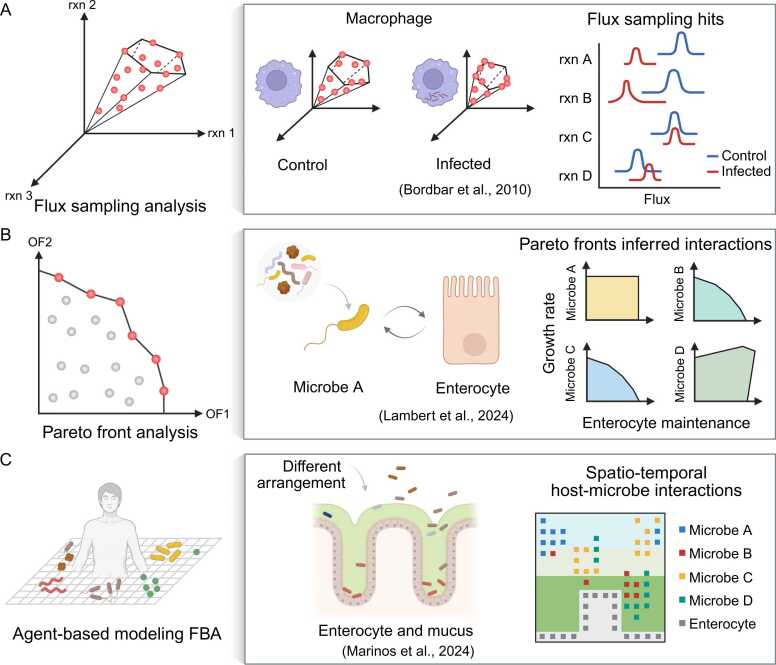
Complementary simulation and analysis approaches for host-microbe GEMs. (A), Flux sampling can be applied to infer host-microbe interactions. (B) Pareto front analysis further explores trade-offs between host and microbial objective functions, revealing shifts in metabolic interactions. (C) Agent-based modeling FBA simulates host-microbe metabolic interactions while incorporating spatial and temporal dynamics. rxn - reaction, OF - objective function.

Another similar approach was introduced with sampling of the solution space of flux distributions through the network [91], [92] (Fig. 3A). Similar to FVA, flux sampling also relies on network constraints and topology to generate biologically meaningful results and can (but does not have to) be employed without the definition of an objective function, thus yields biological insights with fewer assumptions than FBA. However, sampling based analysis have not found many applications in large networks (but see [93]), not least due to problems in convergence and/or performance [94]. For example, Bordbar et al. (2010) applied this approach to compare flux sampling distributions (probabilities) between normal macrophage and macrophages infected with *Mycobacterium tuberculosis* [95]. Another technique is to analyze the Pareto front [96] (Fig. 3B). A Pareto front is a set of optimal solutions in multi-objective optimization problems where no objective can be improved without worsening at least one other. In metabolic modeling, this means identifying metabolic states where trade-offs between competing objectives, such as growth of individuals in a community with limited resources, are balanced. Each point on the Pareto front represents a unique compromise, and together they outline the boundary of feasible, non-dominated solutions that reflect the system's capabilities under given constraints. Though, optimizing bi-objective (or multi-) functions implies direct interactions between organisms (e.g., a host cell and a microbial cell). This approach excludes potential sequential metabolic processes across multiple organisms, as commonly observed in microbial communities. Work by Lambert et al., 2024 demonstrated the use of Pareto front analysis to reveal pairwise interactions between a microbial species and a host enterocyte cell [96]. The shape of the Pareto front can provide insights into potential interaction patterns like mutualistic or competitive relationships (Fig. 3B).

Although not widely used, to model host-microbe interactions an agent-based metabolic modeling framework could offer an alternative by simulating independent microbial and host cells within a spatial grid [97] (Fig. 3C). As an example, Marinos et al. adopted this approach to develop VirtualColon, which simulates interactions between enterocyte cells and microbes within mucus layer gradients. Their results demonstrated dynamic metabolic interactions and the resulting spatial organization of microbes [97]. Unlike traditional metabolic models that require an integrative metabolic network and predefined objective function, the approach allows individual organisms to optimize their own metabolism, whether through maximization of biomass or ATP biosynthesis, and interact dynamically with the surrounding environment. Within this context, the approach assumes a higher degree of individual independence. Since the metabolic networks of organisms are simulated independently and remain unconnected, costly metabolites are unlikely to be secreted and shared; instead, metabolic byproducts or wastes are more likely to accumulate. Further constraints, e.g., resource allocation, limit the distribution of a cellular resource, such as total protein, energy, or flux [98], [99], may alleviate the assumption but their effectiveness in the agent-based modeling FBA still requires further proof of concept. This approach faces further challenges, including high computational demands and the need for additional parameters such as rules of reproduction and death, movement patterns, metabolite diffusion rates, and grid size, which require substantial prior knowledge for proper implementation.

A major obstacle in all these simulations of host-microbe interactions is the lack of a suitable objective function for eukaryotic cells, as their objective function is either ill-defined for modeling or simply not known. It is even debatable if a single objective function for individual cells in multicellular eukaryotes exists, given the changing metabolic demands which are imposed by the interplay of different tissues. For most non-model organisms, the biomass function is not even known and must be derived from experimental data or literature values. BOFdat has tried to standardize this procedure at varying levels of detail (from macromolecular measures to detailed co-factor production) [100]. Current studies often use “maintenance” as an objective in eukaryotic cells, yet this usually represents a reduced biomass objective function and does not mirror actual cellular functions in a multicellular organism [27], [96]. Thus, modeling approaches currently remain restricted to well defined systems. However, there have been some approaches to circumvent these shortcomings. First, the integration of transcriptomic or proteomic data led to the development of algorithms like RegrEx [101], SPOT [102], or MOOMIN [103] which minimize the distance of reaction flux to enzyme expression or differential change in expression. Hence these algorithms provide a flux distribution without optimizing an objective function. Other tools have used transcriptomic or fluxomic data to determine the individual cellular objective in a group of cells from the same organism. SCOOTI [104] tries to infer individual cell objectives from multicellular tissues by fitting gene expression to flux vectors of different predefined objectives. ObjFind [105] uses a similar approach by optimizing fluxes through the network to match those measured in fluxomic experiments. In combination with the FBA approaches for microbial community models, these different approaches could be a valuable alternative to model host microbe interactions. Another alternative would be (semi-) dynamic models which use ordinary differential equations to estimate metabolic flux through the metabolic network by reaction rates, enzyme and metabolite concentration for each reaction. However, the many parameters required are still a major hindrance to model metabolism at the cell level [106]. However, hybrid models have been developed like ECMpy [107], sMOMENTS [108] or GECKO [109] which consider protein and reaction rate constraints during metabolic modeling to alleviate some of these problems. However, they still require an objective function to estimate flux distribution, but do not solve the problem of inaccurate eukaryotic modeling by FBA. Other approaches use a Petri-Net representation of metabolic models integrating knowledge of reaction rates, metabolite and protein concentrations, which showed promising results on even relatively large scales [110]. Thus combining these approaches could facilitate accurate modeling of eukaryotic metabolism without knowing specific objectives of the modelled cell and could open the door for a large-scale framework of host-microbe metabolic modeling.

## 3 From single-species to microbe-microbe metabolic modeling

The initial study of GEMs was conducted to investigate metabolic capacity of the pathogenic bacterium *Haemophilus influenzae* by Edwards and Palsson [111]. Over time, this modeling approach has advanced by incorporating additional features across multiple dimensions, including community interactions, space and time [80], [112], [113], [114]. Microbial communities are hereby treated like a highly compartmentalized tissue, where each bacterial species forms an ‘organelle’ able to interact with each other. Together with adaptations in the methodology to reliably simulate flux through the community, we can predict metabolic interactions between microbes [114], [115]. Furthermore, the integration of multi-omics (e.g. metagenomics, metatranscriptomics and metabolomics) [67], enzyme kinetics [116], and thermodynamic constraint [117], alongside with the development of computational approaches e.g. individual-based modeling [80] and machine learning approaches, e.g. for the prediction of metabolic flux and enzyme turnover [118], [119], [120], has further expanded utilization of this modeling approach. These developments have been rapidly expanded to a wide range of research fields, including biotechnology, bioremediation, environmental microbiome, medicine, including host-microbe interactions [121], [122].

Various studies across different host contexts for example, in humans and in *C. elegans*, have adopted metabolic modeling approaches to investigate host-microbe interactions. These studies have relied on using either a host model or a microbial community model in isolation, without fully integrating both systems. To approximate host-microbe conditions, contextualizations of models are usually conducted by integrating additional layers of data such as transcriptomics or metagenomics. For instance, Taubenheim et al. (2025) constructed personalized microbial community GEMs contextualized with amplicon data, together with human GEMs contextualized by bulk transcriptomic data from colon biopsies and blood samples of patients with inflammatory bowel disease (IBD). Their models revealed that IBD patients exhibited reduced metabolic activity in both the microbial community and host tissues, particularly in processes such as NAD recycling and synthesis between host and microbe [123]. Similarly, a study of the symbiosis between *C. elegans* and *Pseudomonas fluorescens* MYb115 integrated the worm’s metabolic model with transcriptomic data collected during infection with *Bacillus thuringiensis*. This approach highlighted host-microbe metabolic re-routing as a protective strategy against infection [66]. Reversily, metabolic modeling of *Escherichia coli* was used to understand how mutants for the cytochrome oxidase cause developmental delay in *C. elegans* [124]. Similar strategies were commonly adopted among modelers to unravel associations of metabolite production, ecological interactions or pathway abundance with different host phenotypes, like disease [125], [126], [127], [128], [129], [130], [131].

While such a strategy yields valuable insights, they underestimate the impact of reciprocal interactions between host and microbes, which are inherently complex and could therefore limit a comprehensive understanding of the overall system [132], [133], [134]. Hence, an integrative approach including the host metabolic functions should be considered to get to a holistic understanding of these interactions.

## 4 Study of host-microbe interaction using host-integrated microbial community metabolic modeling

**Fig. 4:**
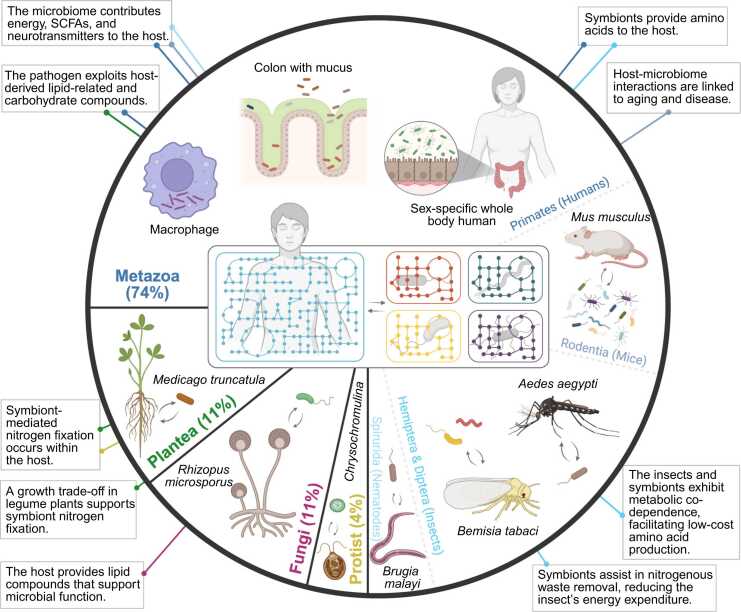
Integrated metabolic modeling in host-microbe interaction studies. Host-microbe metabolic models have been applied across diverse host systems, including Metazoa (across taxonomic orders: Primate (e.g. human), Rodentia (e.g. *Mus musculus*), Hemiptera and Diptera (e.g. *Bemisia tabaci* and *Aedes aegypti*), and Spirurida (e.g. *Brugia malayi*)), Plantea (e.g. *Medicago truncatula*), Fungi (e.g. *Rhizopus microsporus*) and Protist (e.g. *Chrysochromulina tobin*). These models have revealed both host-specific interactions (e.g. nitrogenous waste removal in insects) and shared mechanisms across systems (e.g. symbiont-driven nitrogen assimilation in legumes and microalgae; pathogen exploitation of lipid and carbohydrate metabolism in tomato and humans). The proportion for each host group represents the relative amount of work devoted to each, to the best of our knowledge, as accounted for in this review.

To date, only few studies exist where host and microbial modeling have been integrated in a single modeling approach (Table S1). Although, these works revealed the power and detail to advance biological knowledge and to generate hypotheses for practical applications. Host-microbe metabolic modeling helped understanding bidirectional interactions, providing a systems-level perspective on how microbial metabolism influences host processes and vice versa within the dynamic context of the environment (Fig. 4). Their applications span a wide range of host systems, including Metazoa (across taxonomic orders: Primate, Rodentia, Hemiptera, Diptera, and Spirurida), Plantea, Fungi, and Protist.

### 4.1 Metazoa

#### 4.1.1 *Primate*s (humans)

The host associated microbial community plays a crucial role in human health, influencing digestion, immunity, and disease susceptibility. Accumulating evidence further indicates that microbes are linked to diverse processes of human physiology, such as metabolism and circadian rhythms, as well as to behavioral states, including depression and social functioning [135]. Host-microbe integrated GEMs have been used to study various aspects, including pathogenesis, the effects of probiotics, and the general association of microbial communities with host health. A pioneering study by Bordbar in 2010 integrated a human alveolar macrophage model with a model of the pathogenic bacterium *Mycobacterium tuberculosis* to investigate tuberculosis pathogenesis [95]. A FVA and flux sampling approach revealed that macrophages derived metabolites, such as hyaluronan supported *M. tuberculosis* growth, as well as vitamin D and folate which promoted disease progression. The results were validated by comparison with literature reports. Later work building on these results evaluated potential metabolic pathogenicity mechanisms and predicted potential therapeutic interventions in tuberculosis [136]. Several metabolically active drugs e.g. TMC207, BTZ043, cycloserine, ethambutol, ethionamide, propionamide, and isoniazid were proposed and described disrupting pathogenic processes and host-pathogen interactions, thereby limiting the growth of *M. tuberculosis*.

An integrative GEM approach using FVA studied the beneficial bacterium *Enterococcus durans* and its metabolic roles in supporting silver nanoparticle (AgNP) treatment for colorectal cancer (CRC). It successfully identified potential novel drug targets and results were verified using literature reports [88]. A similar study analyzed the influence of high fiber diets on the interactions of *Lactobacillus rhamnosus* and CRC cell cultures *in-silico* and *in-vitro.* Here, modeling predicted and experiments confirmed that an increase in short-chain fatty acid production by *Lactobacillus* combined with the competition for amino acids between bacteria and host caused a significant reduction in pro-oncogenic gene expression and attenuated self renewal of CRC cells [137]. A recent study integrated host and microbial modeling to study inflammatory bowel disease patients. This approach revealed that patient-specific microbial communities increased amino acid catabolism and vitamins like thiamine and folic acid. Further, an increase in *Desulfovibrio piger* has direct consequences on liver sulfur metabolism of patients [138].

Further work of microbe-host metabolic modeling has revealed the role of co-metabolism in Alzheimer's disease [139], [140]. FBA demonstrated that, in addition to host genetics, microbially derived metabolites, such as glucose, L-tyrosine, and L-tryptophan affect host formate biosynthesis, thereby influencing urinary formate concentration, which may serve as an indicator of onset of Alzheimer’s disease [139]. Another study has shown that the bile acid deoxycholate, produced by *Eggerthella lenta*, is positively associated with the APOE gene, a well-known risk factor for Alzheimer’s disease, suggesting a potential role of the microbiota in the disease [140]. The result was later validated by blood serum metabolomics. Furthermore, human gut microbial communities, ranging from a few to over hundred species in personalized communities, have been modeled with human cells to investigate their ecological roles in host health [26], [27], [96]. With Pareto optimality, metabolite exchange analyses, and abundance weighted biomass FBA, these studies have highlighted the significant benefits of the microbial community in supporting host metabolism by supplying essential amino acids [26], neurotransmitters, and energy compounds to the colon [27]. Notably, choline secretion by *L. rhamnosus* GG was predicted to contribute to enterocyte physiology and the colonic ecosystem in general [96]. A recent spatiotemporal agent based simulation demonstrated niche modification in the colon, where the mucus-degrading bacteria, *Bacteroides thetaiotaomicron* alters the layered mucus and enables access for other microbes [97].

Apart from host-microbe studies, metabolic modeling has also been applied to host-parasite interactions, such as between the malarial parasite *Plasmodium falciparum* and human red blood cells (RBCs) [141]. The host-parasite metabolic model was used to capture different life-cycle stages of *P. falciparum*, which relies on RBC metabolism. Simulations revealed that uninfected RBCs coexisting with infected cells adapted their metabolism by reducing the production of 2,3-bisphosphoglycerate, a regulator of hemoglobin oxygen binding. Furthermore, predictions of metabolite uptake and secretion by the host-parasite model showed strong agreement with experimental metabolomic data, supporting the reliability of the model’s predictions.

#### 4.1.2 Rodentia (mice)

Mice are widely used as animal models for microbiome studies in medical research due to their genetic similarity to humans, well-established experimental and manipulation techniques, and practical advantages such as short life cycle, small size, and relative affordability [142], [143], [144]. In mice, the resident microbial community shows similarity to the one in humans and plays a crucial role in key physiological processes, such as immunity and metabolism [142], [145]. Also, the availability of synthetic microbial communities like the Altered Schaedler Flora (ASF) [146], the extended simplified human microbiota (SIHUMIx) [147] and the oligo mouse microbiota (OMM12) [148] to colonize mice, allows researchers to study host-microbe interactions with greater precision on microbial genomic-scale analysis or manipulation.

GEMs have been applied to mouse-microbe systems to infer the effects of host-microbe interactions on host health. One study investigated the ecological interactions between *B. thetaiotaomicron* and mouse models under different dietary regimes [149]. FVA and Pareto front analysis revealed that the presence of *B. thetaiotaomicron* facilitated the breakdown of complex polysaccharides into smaller sugar molecules, which were subsequently absorbed by the host. However, while a mutualistic relationship was observed, excessive growth of *B. thetaiotaomicron* was found to reduce the host’s maximum growth rate. The same mouse-*B. thetaiotaomicron* system was also used to reveal metabolic interactions via outer membrane vesicles (OMV) [90]. FBA and metabolic exchange analysis suggested that *B. thetaiotaomicron* selectively packed OMV metabolites which are transferred to the mouse-host are preferred and enhance the mouse metabolic capabilities.

In the context of aging, a recent study used extensive multi-omics data and integrated this into a host-microbe metabolic model of mice to understand changes in metabolic interactions during aging. The authors used abundance weighted biomass FBA, FVA, and elementary flux mode sampling to show a pronounced loss of microbial metabolic activity with age leading to a concomitant loss of host-microbe interactions with age. Interactions between microbial species shifted from mutually beneficial to more competitive interactions among each other with increasing age. At the same time the lack of microbial metabolic activity in SCFA and nucleotide production led to alterations in colon, liver, brain functions associated with aging [89].

#### 4.1.3 Hemiptera and Diptera (insects)

Symbiotic relationships between insects and microorganisms are well recognized. Certain insects, particularly those adapted to nutrient-poor diets such as plant sap and wood, have evolved a specialized organ, the bacteriome, to host essential genome-reduced bacteria that are vertically transmitted from parent to offspring [150], [151]. These bacteria sustain their host’s health by providing essential nutrients, while the insects reciprocate by supplying compounds such as amino acids and vitamins [151]. Metabolic models of host-microbe interactions in insects, especially in the order Hemiptera, and their symbionts have been used to capture complex interactions that are otherwise difficult to observe. For instance, modeling the whitefly *Bemisia tabaci* and its associated microbes, *Candidatus Hamiltonella defensa* and *Candidatus Portiera aleyrodidarum*, has revealed mutualistic interactions [85]. Simulations demonstrated that *B. tabaci* utilizes its microbial symbionts as metabolic units, supplying precursors and subsequently deriving essential amino acids for its own use. Furthermore, *C. Portiera* and *C. Hamiltonella* assist in removing nitrogenous waste from the host thus potentially reducing further energy required for nitrogen disposal. Similarly, in other insect-microbe systems such as cicada *Neotibicen canicularis* (symbionts: *Hodgkinia* and *Sulcia*); sharpshooter *Graphocephala coccinea* (symbionts: *Baumannia* and *Sulcia*); spittlebugs *Philaenus spumarius* (symbionts: *Sodalis* and *Sulcia*) and *Clastoptera proteus* (symbionts: *Zinderia* and *Sulcia*); and aphids *Acyrthosiphon pisum* (symbionts: *Buchnera* and *Hamiltonella*), metabolic models have indicated metabolic co-dependence of hosts and microbiota. Optimizing overall amino acid production via FBA showed that insect hosts derive amino acids from their symbionts, indicating that this loss of metabolic function was outsourced to the partner to reduce metabolic costs [86], [152], [153], highlighting the evolutionary significance of these relationships. The modeling was confirmed by ¹ ³C isotopic profiling, which showed that energetically expensive essential amino acids are collaboratively synthesized and shared with the host by *Sulcia* and *Zinderia* in spittlebugs [152]. Hence, the application of metabolic modeling in the context of insect-microbe interactions provides valuable insights into the coevolution of hosts and their symbionts, revealing adaptive strategies that enhance survival in nutrient-limited environments.

In addition, a metabolic model of symbiotic systems between a mosquito (*Aedes aegypti*) and *Wolbachia pipientis* was reconstructed to investigate its potential role in Dengue control [154]. FBA simulation identified key metabolites, such as isopentenyl diphosphate, tryptophan, valine, methionine, and leucine, transferred from the mosquito host to *Wolbachia*, consistent with previous experimental findings. The host-microbe model was further extended to represent a Dengue-infected state, enabling predictions of the symbiont’s pathogen-blocking potential. The results highlighted that under conditions of high *Wolbachia* growth, competitive uptake of amino acids could impair viral growth, thereby blocking pathogenicity.

#### 4.1.4 Spirurida (nematodes)

Next to evolutionary insights, host-microbe weighted biomass FBA modeling has been used to suggest new therapeutic targets for parasite infections. The intracellular symbiont *Wolbachia* supports the survival of its parasitic host *Brugia malayi* by exporting ATP under low-oxygen conditions and buffering shifting carbon availability [84]. This dependence of the host from its symbiont was used to suggest and in-vitro validate metabolic reactions which served as novel drug targets to disrupt parasite viability.

### 4.2 Plantea

Microorganisms and plants form intricate mutualistic relationships that are fundamental to nutrient exchange, growth promotion, and disease processes, making them critical in agricultural and environmental sciences [155]. GEMs have been used to study the symbiosis between the legume plant *Medicago truncatula* and the nitrogen-fixing bacterium *Sinorhizobium meliloti*. The first model of this interaction was developed by Pfau et al. (2018) [156] and later refined by diCenzo et al. (2020) [83]. Abundance weighted biomass FBA of these models revealed the primary carbon and nitrogen sources (sucrose and ammonia) that are supplied from *M. truncatula* to *S. meliloti*. Simulation results demonstrated a trade-off between growth and nitrogen fixation in the host. The result indicated that the symbiosis can slightly reduce plant growth rates due to the metabolic costs associated with biosynthesis of building blocks of the symbiont, however, this trade-off is less severe compared to scenarios with insufficient nitrogen fixation. This emphasises the net benefits of the mutualistic relationship to the host plant.

In a plant pathogenic context, the GEM of the fungal-like eukaryotic microorganism *Phytophthora infestans* interacting with its host, tomato (*Solanum lycopersicum*), provided insights into its parasitic strategies [72]. Biomass weighted FBA indicated that *P. infestans* predominantly relies on host-derived amino acids and lipid-related molecules for biomass synthesis. Furthermore, the model suggested that targeting specific metabolic host-pathogen interactions, such as those involved in host thiamine biosynthesis, could serve as a novel strategy for disease management.

### 4.3 Fungi

Bacterial endosymbionts within fungal hosts have been reported to influence growth, metabolism, and reproduction [157]. A recent study by Valadey-Cano et al. (2024) employed a host-microbe metabolic model to investigate the holobiont interactions among *Rhizopus microsporus* (fungal host), *Mycetohabitans* (bacterial endosymbiont), and *Narnavirus* (virus) [158]. FBA and FVA of the holobiont model revealed that the fungal host shared lipid compounds, specifically glycerol, with the bacterial symbiont, consistent with previous experimental evidence. In return, *Mycetohabitans* produced rhizoxin, an antimitotic agent that facilitates the parasitic lifestyle of *R. microsporus* in plants. Moreover, the viruses were shown to upregulate nucleotide metabolism in the fungal host by increasing the production of nucleotide precursors.

Beyond endosymbiosis, fungal-bacterial relationships have also been studied with integrated GEMs in the context of symbiosis with higher eukaryotic hosts, for example in humans and poultry [159], [160]. For instance, a previous study investigated the interactions between *Candida albicans*, a commensal but opportunistic member of the human gut microbiota, and various bacterial species [159]. Pairwise simulations showed that co-growth with *Alistipes putredinis* suppressed the growth of *C. albicans*, pointing to a potential therapeutic strategy for future experimental validation. In poultry, fungal-bacteria GEM simulations indicated that fungal genera such as *Clavispora*, *Aspergillus*, and *Saccharomyces* have positive interactions with the poultry microbial community [160]. To validate model quality, predictions of SCFA production across different ages and gut sections of chickens were compared with experimental measurements, which showed good agreement.

### 4.4 Protist

Apart from multi-cellular eukaryotes, host-microbe metabolic modeling was also adopted in a unicellular photosynthesis haptophyte, *Chrysochromulina tobin* *[*161]. In marine ecosystems, *Candidatus Atelocyanobacterium thalassa* fixes nitrogen for its algal host *C. tobin*, a process incompatible with oxygen producing photosynthesis. The modeling revealed that this nitrogen supply occurs in exchange for several carbon sources, vitamins and amino acids, that the amount of nitrogen fixation is dependent on the carbon source provided, and suggested that “Mehler’s reaction” is the most efficient way to scavenge free oxygen in the symbiont [161]. These host-microbe GEM studies underscore how microbial symbionts shape host metabolism and influence ecological interactions.

## 5 Summary and outlook

Metabolic modeling offers insights into metabolic exchanges and community functions of host-microbe interactions and has been applied across a wide range of host organisms, including Metazoa (Primate, Rodentia, Spirurida, and Hemiptera and Diptera), Plantea, Fungi, and Protist. These models have been used in systems ranging from simple host-microbe pairs to complex microbial communities with over 100 species and have provided valuable insights into mechanisms of disease pathogenesis, therapeutic strategies, host-microbe functional relationships under different environmental conditions and more. Exchange of amino acids, lipids, vitamins and energy metabolites have thereby emerged as a common hallmark of mutualistic relationships, often accompanied by some sort of nitrogen fixation or waste removal. This is in contrast to pathogenetic/parasitic relationships, where microbes use a wide variety of metabolites to enhance their own growth at the expense of the host. With technical advancements in the field, metabolic modeling has become more accessible due to (semi-) automated reconstruction of GEMs, integration of omics data, and convenient simulation and analysis tools.

However, many technical challenges regarding host-microbe metabolic modeling remain unsolved. This starts with model reconstruction of host (eukaryotic) models, as compartmentalization, metabolite transport and metabolic capabilities are inherently more complex in eukaryotes compared to microbial models [162]. This is associated with an expansion of metabolic genes and the diversification of regulation of metabolism [163], which makes it difficult to properly reconstruct eukaryotic GEMs. Current eukaryotic reconstruction frameworks depend on homology searches either based on databases [38], [44], [45], [47] or on reference models [48]. However, homology of individual enzymes and transporters might be obscured by long evolutionary divergence, structure-based homology search could alleviate these limitations [164]. AlphaGEM already incorporates structural information and additionally uses AI tools to infer homology and metabolic functions from eukaryotic genomes [49]. Eventually, all of these tools will provide a draft metabolic model, which needs to be gapfilled to connect loose ends in the metabolic network. Here, various tools exist with different assumptions, considering for example fluxomics data, gene-co-expression, or evidence of homology (see Review for details: [165]). Furthermore, it is advisable to contextualize the GEMs to represent the condition under study. The integration of transcriptomic and metabolomic data is a widely used approach for this purpose, and several tools have been developed to generate context-specific models [166]. Depending on the modeling scenario, applying these techniques to various tissues/organs of the host allows studying metabolic interactions between different organs and probing their dependence on the microbiota [27], [89]. These multi-tissue or organ-compartment models have been highly informative for understanding complex metabolic interactions such as those underlying the gut-bain axis [89] and should be applied to other (non-) model systems as well. In a similar sense, but on a smaller scale, single cell RNAseq allows for the reconstruction of cell specific metabolic models [64], [65]. This opens up the possibility of micro-scale spatial models investigating the direct interaction between microbes and different cells in the epithelium. Marinos et al. [97] and Lambert et al. [96] show how such a modeling approach with single cells could generally look like.

Constructing the host model represents a substantial effort, yet integration of models also needs consideration when modeling host-microbe interactions. Microbial models are often either derived using one of the reference databases [34], [35] or are reconstructed from genomic information [39], [40] while host models are often derived from other source like VMH [167] (compatible with AGORA2), the metabolic atlas [168], ModelSEED [37], [38] (compatible with gapseq) or BiGG [35] (compatible with CarveMe). All these resources use different namespaces and variants of metabolites, which complicate merging models of different origin. While a simple name conversion table for metabolites is relatively easily derived (see MetaNetX [56]), finding the same metabolite variants between two models is more difficult. Often, the models lack structural information for the metabolites they use and names are inconsistent between different databases. Further, different databases sometimes represent the same metabolites inconsistently, using different protonation states, isomeric forms, polymer lengths, or even lumping groups of metabolites under a single common name. Merging inconsistent models can lead to thermodynamically impossible cycles and stoichiometric imbalance models. In these cases, simply cycling inconsistent metabolites can artificially generate ATP (or other metabolites) from nothing. Future efforts and tools should be directed towards the synchronization of metabolite databases and models to facilitate automatic merging of models between an eukaryotic host and microbial community.

Once the models are merged, the analytic framework needs to be considered. To define objective functions for host models is difficult on its own, yet deriving an integrated one together with the microbial community can become even more difficult. This is often only realistic, if the modeled system is well known and a common metabolic goal can be defined, as in the case of the production of amino acids in the hemiptera studies [85], [86], [152], [153]. Most of the time, a specific objective is unknown and instead weighted biomass production was optimized as a measure of fitness of the holobiont. However, this assumption falls short on at least two levels: (i) it requires that the host is also actively growing or accumulating biomass, which is often not the case, and (ii) growth is not necessarily a reliable proxy for fitness, as resource allocation differs across organisms and modes of reproduction [169]. Further, the balance between biomass contributions of the individual models to overall growth has a direct impact on the modeling outcomes, making it a subject of study via Pareto front analysis [96]. To circumvent these problems many studies used objective free analysis of flux distributions, like FVA, flux sampling and metabolite exchange analysis. However, these approaches tend to overestimate the metabolic interactions between host and microbes, as they analyse the models for all possible interactions and not only the most likely ones. Taken together, this constraint currently hinders straight-forward host-microbe model analysis, underscoring the need for careful interpretation and limiting its applicability. New integration tools to define objective functions [104], [105] or derive flux distributions [101], [102], [103] might be able to solve these problems, yet require more input data. On the other hand, dynamic modeling frameworks like COPASI [170] can be a solution in the future. Especially given that previously hard to derive parameters as reaction rate constants are being more reliably predicted with modern machine learning and AI tools [171], [172], [173], [174], [175], [176], [177]. Generally, as models expand and become more complex, computational costs become a limiting factor. To address this issue, the idea of reduced models has been formulated, where the metabolic model is reduced to the net-conversion rates of metabolites, omitting all (or many) internal reactions [178], [179], [180]. By reducing the individual models, the approach reduces not only the computational costs but also excludes unrealistic metabolic conversions [179]. It further opens up the possibility to tie metabolic modeling into ecological modeling of consumer-resource models [181], [182], facilitating the analysis of large scale bacterial dynamics in host context.

One intrinsic characteristic of metabolic modeling is its focus on metabolism as a single explanatory layer. While this perspective often captures the downstream consequences of other biological processes and is frequently close to the expressed phenotype, studying metabolism alone does not disentangle additional layers of interaction that are particularly relevant in host-microbe systems, such as epigenetics, signalling, and physiological processes [183], [184], [185], [186], [187]. Covert et al. (2008) combined metabolic models with gene regulatory networks using Boolean logic and ordinary differential equations [188]. These studies highlight the flexibility and versatility of metabolic modeling, where metabolism is used as a foundation onto which additional layers of biological regulation can be systematically integrated. However, greater integration also requires more extensive datasets, increases model complexity, and introduces additional assumptions. Careful interpretation is therefore essential, particularly when multiple layers of interaction are involved. Despite these challenges, GEMs remain powerful tools with strong potential to advance our understanding of host-microbe systems.

There is potential of metabolic host-microbe modeling to address current pressing problems in ecology, evolution, agriculture and medicine. For example, it could help to understand and mitigate diversity loss, due to climate change and habitat loss. A prime example in this regard is coral bleaching, induced by increased water temperatures and causing the loss of one of the most diverse habitats on earth [189], [190]. Bleaching occurs when the coral expels their photosymbionts, which eventually lead to death of the coral. Interestingly, this phenomenon seems to be triggered by the loss of metabolic control of the host over the photosymbiont [191], [192]. Interestingly, the effects of coral bleaching have been shown to be dependent on the microbial composition [193], with metabolic functions playing key roles in this interaction [194]. High quality metabolic models could contribute to understanding these complex relationships and how to establish more heat tolerant holobionts to mitigate the bleaching events, for example by probiotics [195]. First, exploratory studies have described metabolic models for microbial coral and sponge communities [196], [197], heading in the right direction. Moreover, host-microbe interactions are important for adaptation and speciation, where metabolic modeling could contribute to understanding evolutionary trajectories. Microbiomes have been shown to play important roles in the adaptation to rapidly changing environments [198] and pose important factors for evolutionary concepts that go beyond direct genetic heritage [199], [200]. Here, we anticipate great potential of host-microbe modeling to delineate evolutionary constraints and trajectories that are found in the metabolic cross-talk in the metaorganism. Some of these concepts have been explored in microbial community modeling [201], yet studies for host-microbe interactions in this regard are still underexplored.

Agriculture and land use are major drivers of climate change and biodiversity loss. Efficient land and water use, along with crops resistant to drought and pathogens, are therefore crucial. Bacteria can induce resistance to drought [202], [203] and pathogens [204], [205]. Metabolic modeling offers tools to study microbial contributions to plant health and to guide the design of beneficial microbiota. First applications include modeling rhizosphere communities in apple trees [206] and drought responses in a rice GEM [207], with further above mentioned examples in tomato and *Medicago trunculata* showing the value of host-microbe modeling [72], [83], [156]. Husbandry is another field in agriculture where problems can be addressed using metabolic modeling. While rumen microbiota has long been studied [208] metabolic modeling is only beginning to be applied [209], promising both methane reduction and better nutrient use. In aquaculture, metabolic modeling has been used to improve salmon growth [210], [211], though microbial integration is lacking. In poultry, fungal-bacterial microbiota models highlight fungal roles, but the host remains excluded [160]. To date, livestock other than cattle, salmon, and poultry remain largely untouched by metabolic modeling, leaving great potential for improving feeding, rearing, and breeding strategies through a systems view of the animal holobiont.

Finally, metabolic modeling of host-microbe interactions has great potential in medical contexts where it can be used to identify underlying mechanisms of complex pathologies with microbial involvement and to propose personalized therapies to treat these diseases. These applications are starting to emerge proposing dietary interventions [123], synthetic communities for treatment [212], or predict conditions for obligate infections [213], [214]. However, host integration is still rare and is just starting to emerge as a way to understand the host influence in microbial dynamics.

Metabolic modeling of host-microbe interactions is rapidly advancing, but significant challenges remain in data integration, model standardization, and computational scalability. By combining emerging omics technologies with improved modeling frameworks, the field is well-positioned to deliver deeper insights into host-microbe systems. Ultimately, these efforts will not only enhance our mechanistic understanding but also open new avenues for translational applications in health, agriculture, biotechnology, and environment.

## CRediT authorship contribution statement

**Natchapon Srinak:** Writing – review & editing, Writing – original draft, Visualization, Conceptualization. **Florian Krüger:** Writing – review & editing. **Christoph Kaleta:** Writing – review & editing, Funding acquisition. **Jan Taubenheim:** Writing – review & editing, Supervision, Funding acquisition, Conceptualization.

## Declaration of Competing Interest

The authors report there are no competing interests to declare.

## Glossary

Constraint-based modeling: A framework that uses stoichiometric, thermodynamic, and environmental constraints to define feasible flux distributions in a metabolic model.
Metabolic network: A representation of the biochemical reactions and metabolites, typically showing how metabolites are transformed and connected through pathways.
Stoichiometric matrix: A mathematical encoding of the metabolic network, where rows represent metabolites and columns represent reactions, with entries indicating stoichiometric coefficients.
Objective function: A mathematical function (e.g., maximizing biomass growth) that defines the optimization goal of the model.
Steady-state assumption: The assumption that metabolite concentrations remain constant (input equals output) during simulation.
Constraints: Boundaries that restrict the solution space (e.g., nutrient availability, enzyme capacity, or uptake limits).
Genome-scale metabolic model (GEM): A computational model that expands the metabolic network into a genome-annotated, stoichiometrically balanced reconstruction. GEMs include reaction directionality, constraints and biomass composition, enabling quantitative simulations.
Flux balance analysis (FBA): An approach that predicts metabolic fluxes by linear optimization of an objective function under steady-state assumption and applying known constraints.
Steady-state assumption: The assumption that metabolite concentrations remain constant (input = output) during simulation.
Solution space: The set of all feasible flux distributions under the given constraints.
Flux: The rate at which metabolites are converted by a reaction in the metabolic network, typically expressed as mmol per gram dry weight per hour (${\mathrm{mmol}\cdot h}^{-1}\cdot g_{\mathrm{dry weight}}^{-1}$)
